# *Aedes taeniorhynchus* in urban areas of northern Peru: a concerning finding in the context of the coastal El Niño phenomenon 2026

**DOI:** 10.17843/rpmesp.2026.432.15787

**Published:** 2026-06-04

**Authors:** Alfredo Julian Sandoval-Norabuena, Ricky Alonso Chumacero-Correa, Raul David Izquierdo-Ramos, Sararosa Victoria Martínez-Alvarado, Cristian Alejandro Lopez-Dominguez, Archi Alejandro Ruiz-Polo

**Affiliations:** 1 Escuela Profesional de Ciencias Biológicas, Facultad de Ciencias, Universidad Nacional de Piura, Piura, Peru.; 2 Escuela Profesional de Biotecnología, Facultad de Ciencias, Universidad Nacional del Santa, Nuevo Chimbote, Peru.; 3 Programa Académico de Enfermería, Facultad de Ciencias de la Salud, Universidad Tecnológica del Perú, Piura, Peru.

Dear Editor. The expansion of mosquitoes is due to their great adaptability and external factors such as climate change, globalization, and urbanization, which facilitate their dispersion in various regions ^1^. *Aedes taeniorhynchus* is a mosquito from tropical and subtropical areas that transmits encephalitic arboviruses; furthermore, its larvae develop in brackish coastal environments and, during the rainy season, the species can expand to rural and urban areas ^2^. However, it can also survive in high concentrations of ammonia, such as those found in wastewater, sewage, or septic tanks, which further expands the potential habitats available for this species and not for others without evolved mechanisms to resist both salt and ammonia simultaneously ^3^.

According to the World Health Organization, in 2021, over 2 billion people resided in countries with water scarcity, a problem exacerbated by climate change and population growth ^4^. According to a report published in 1961, Peru also faces this problem, as departments such as Lambayeque, Piura, and Tumbes have recorded limitations in water access and associated effects, such as increased salinity, for several decades ^5^. Currently, this problem has worsened in Piura due to its vulnerability to climatic phenomena, such as El Niño Costero, as well as sustained population growth, which has increased dependence on water supply through subterranean wells, whose waters present various contents of salts and ammonia. In this context, it is important to study the distribution of *Ae. taeniorhynchus* in Piura, as it can go unnoticed and presents adaptations to diverse environmental conditions.

**Figure 1:**
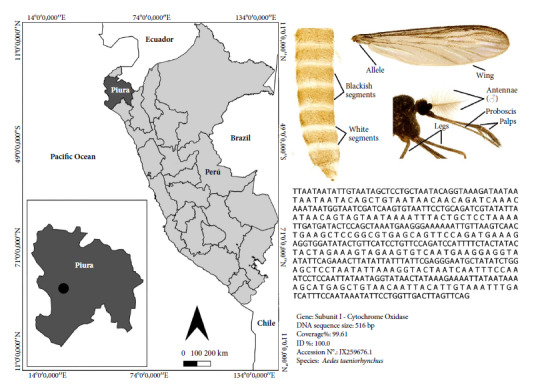
Phenotypic and genotypic record of *Ae. taeniorhynchus* in an urban cemetery in the department of Piura, Peru in 2025. The black circle shown on the map of Piura indicates the location of the urban cemetery evaluated in the Catacaos district. The nitrogenous base sequence belongs to the coding fragment of the COI gene of the species *Ae. taeniorhynchus*. The images presented show characteristic morphological features of the species’ phenotype.

A study was conducted in April 2025, where adult specimens of *Ae. taeniorhynchus* were collected in the general cemetery of Catacaos (5°15’57.35’’S / 80°40’14.52’’W), located in the town of the same name (Figure 1), which has a hot and mostly dry climate with periods of intense precipitation. The capture method consisted of applying the resting capture technique. Specimen identification was performed in the microbiology laboratory of the Universidad Nacional de Piura using taxonomic keys ^6^ and, complementarily, by genetic analysis through polymerase chain reaction (PCR) technique, amplifying the mitochondrial COI gene with primers LCO1490 and HCO2198. The protocol included a pre-denaturation at 95 °C for 5 min, followed by 35 cycles composed of denaturation at 95 °C for 30 s, hybridization at 58 °C for 30 s, and extension at 72 °C for 1 min. Finally, an extension at 72 °C for 5 min was performed and products were stored at 4 °C for 24 h. Amplified products were sequenced by the Sanger method in double strand. Then, sequences were aligned to generate consensuses and compared using BLAST with homologous sequences from GenBank.

Regarding the results, no eggs and larvae of the species were recorded; however, three adult specimens were identified that showed tarsal segments with white rings, a clear ring in the middle position of the proboscis, white transverse bands on the abdomen, wings with dark halteres, and a clypeus characteristic of the species (Figure 1). However, considering the unusual presence of adults, the absence of eggs and larvae, a complementary genetic identification was necessary, which allowed confirming that it was the species *Ae. taeniorhynchus* (Figure 1). The record of *A. taeniorhynchus* in a cemetery in Catacaos is relevant and concerning, since its presence indicates a potential epidemic risk and raises hypotheses about its arrival, either by involuntary transport of adults or by eggs that hatched under favorable conditions.

The research had limitations, since due to economic and time restrictions, specimen collection was carried out only within the cemetery and not in nearby areas, which would have allowed for better determination of mosquito movement. Even so, the presence of *Ae. taeniorhynchus* was confirmed outside its typical habitat, evidencing its ecological response to abiotic conditions that favor its displacement.

In conclusion, Piura presents rapid demographic growth that aggravates problems of water access and floods due to insufficient urban planning, which increases the population's health risk against diseases such as dengue and favors the presence of vector species, such as *Ae. taeniorhynchus*. It is recommended to strengthen entomological surveillance and vector control strategies, with special attention to cemeteries as risk areas. Likewise, it is suggested to develop culicid surveillance studies, particularly in the context of the 2026 Coastal El Niño phenomenon, due to climatic conditions that favor the proliferation and dispersion of these vectors.
