# Supplementary material for: Aedes taeniorhynchus in urban areas of northern Peru: a concerning finding in the context of the coastal El Niño phenomenon 2026
Source: Rev Peru Med Exp Salud Publica. 2026 Jun 4;43(2):278–80. doi: 10.17843/rpmesp.2026.432.15787 (PMC13459870; doi:10.17843/rpmesp.2026.432.15787)
Supplement: Translation

# Translation: *Aedes taeniorhynchus* en zonas urbanas del norte del Perú: un hallazgo preocupante en el contexto del fenómeno de El Niño Costero 2026

**Authors:** Alfredo Julian Sandoval-Norabuena, Ricky Alonso Chumacero-Correa, Raul David Izquierdo-Ramos, Sararosa Victoria Martínez-Alvarado, Cristian Alejandro Lopez-Dominguez, Archi Alejandro Ruiz-Polo

**Affiliations:** 1 Escuela Profesional de Ciencias Biologicas, Facultad de Ciencias, Universidad Nacional de Piura, Piura, Perú.; 2 Escuela Profesional de Biotecnologia, Facultad de Ciencias, Universidad Nacional del Santa, Nuevo Chimbote, Perú.; 3 Programa Academico de Enfermeria, Facultad de Ciencias de la Salud, Universidad Tecnologica del Peru, Piura, Perú.

Sr. Editor. La expansión de los mosquitos se debe a su gran adaptabilidad y a factores externos como el cambio climático, la globalización y la urbanización, que facilitan su dispersión en diversas regiones ^1^. *Aedes taeniorhynchus* es un mosquito de zonas tropicales y subtropicales que transmite arbovirus encefalíticos; además, sus larvas se desarrollan en ambientes costeros salobres y, durante la temporada de lluvias, la especie puede expandirse a áreas rurales y urbanas ^2^. No obstante, también puede sobrevivir en altas concentraciones de amoníaco, como las que se encuentran en aguas residuales, aguas de desecho o fosas sépticas, lo cual amplía aún más los hábitats potenciales disponibles para esta especie y no para otras sin mecanismos evolucionados para resistir tanto la sal como el amoníaco simultáneamente ^3^.

Según la Organización Mundial de la Salud, en 2021 más de 2000 millones de personas residían en países con escasez hídrica, un problema que se agrava debido al cambio climático y al crecimiento demográfico ^4^. De acuerdo con un informe publicado en 1961, el Perú también enfrenta esta problemática, pues departamentos como Lambayeque, Piura y Tumbes registran desde hace varias décadas limitaciones en el acceso al agua y efectos asociados, como el incremento de la salinidad ^5^. Actualmente, este problema se ha agravado en Piura debido a su vulnerabilidad frente a fenómenos climáticos, como El Niño Costero, así como al crecimiento demográfico sostenido, que ha incrementado la dependencia del abastecimiento de agua a través de pozos subterráneos, cuyas aguas presentan diversos contenidos de sales y amoníaco. En este contexto, es importante estudiar la distribución de *Ae. taeniorhynchus* en Piura, ya que puede pasar desapercibido y presenta adaptaciones a diversas condiciones ambientales.

Se llevó a cabo un estudio en abril de 2025, donde se recolectaron ejemplares adultos de *Ae. taeniorhynchus* en el cementerio general de Catacaos (5°15’57.35’’S / 80°40’14.52’’O) ubicado en la localidad del mismo nombre (figura 1), la cual posee un clima caluroso y mayormente seco con periodos de precipitación intensa. El método de captura consistió en la aplicación de la técnica de captura en reposo. La identificación de los especímenes se realizó en el laboratorio de microbiología de la Universidad Nacional de Piura mediante claves taxonómicas ^6^ y, de forma complementaria, mediante análisis genético a través de la técnica de reacción en cadena de la polimerasa (PCR), amplificando el gen mitocondrial COI con los cebadores LCO1490 y HCO2198. El protocolo incluyó una predesnaturalización a 95 °C durante 5 min, seguida de 35 ciclos compuestos por desnaturalización a 95 °C por 30 s, hibridación a 58 °C por 30 s y extensión a 72 °C por 1 min. Finalmente, se realizó una extensión a 72 °C por 5 min y la conservación de los productos a 4 °C por 24 h. Los productos amplificados se secuenciaron por el método Sanger en doble cadena. Luego, las secuencias se alinearon para generar consensos y se compararon mediante BLAST con secuencias homólogas de GenBank.

**Figura 1:** Registro fenotípico y genotípico de *Ae. taeniorhynchus* en un cementerio urbano del departamento de Piura, Perú en el año 2025. El círculo negro mostrado en el mapa de Piura indica la localización del cementerio urbano evaluado en el distrito de Catacaos. La secuencia de bases nitrogenadas pertenece al fragmento codificante del gen COI de la especie *Ae. taeniorhynchus*. Las imágenes presentadas muestran rasgos morfológicos característicos del fenotipo de la especie.

En cuanto a los resultados, no se registraron huevos y larvas de la especie; no obstante, se identificaron tres ejemplares adultos que mostraron segmentos tarsales con anillos blancos, un anillo claro en posición media de la probóscide, bandas transversales blancas en el abdomen, alas con balancines oscuros y un clípeo propio de la especie (figura 1). No obstante, considerando la presencia inusual de adultos, la ausencia de huevos y larvas, fue necesario una identificación genética complementaria, la cual permitió confirmar que se trataba de la especie *Ae. taeniorhynchus* (figura 1). El registro de *A. taeniorhynchus* en un cementerio de Catacaos es relevante y preocupante, pues su presencia indica un potencial riesgo epidémico y plantea hipótesis sobre su llegada, ya sea por transporte involuntario de adultos o por huevos que eclosionaron en condiciones favorables.

La investigación tuvo limitaciones, ya que por restricciones económicas y de tiempo la recolección de especímenes se realizó solo dentro del cementerio y no en áreas cercanas, lo que habría permitido determinar mejor el movimiento de los mosquitos. Aun así, se confirmó la presencia de *Ae. taeniorhynchus* fuera de su hábitat típico, evidenciando su respuesta ecológica a condiciones abióticas que favorecen su desplazamiento.

En conclusión, Piura presenta un rápido crecimiento demográfico que agrava problemas de acceso al agua e inundaciones debido a una planificación urbana insuficiente, lo que incrementa el riesgo sanitario de la población frente a enfermedades como el dengue y favorece la presencia de especies de vectores, como *Ae. taeniorhynchus*. Se recomienda fortalecer la vigilancia entomológica y las estrategias de control vectorial, con especial atención a los cementerios como zonas de riesgo. Asimismo, se sugiere desarrollar estudios de vigilancia de culícidos, particularmente en el contexto del fenómeno El Niño Costero de 2026, debido a las condiciones climáticas que favorecen la proliferación y dispersión de estos vectores.
